# Unicuspid aortic valve concomitant with aortic insufficiency presenting with infectious endocarditis: a case report

**DOI:** 10.1186/s13256-019-2239-9

**Published:** 2019-09-20

**Authors:** Naoko Yuzawa-Tsukada, Toshikazu D. Tanaka, Satoshi Morimoto, Michihiro Yoshimura

**Affiliations:** Division of Cardiology, Department of Internal Medicine, The Jikei University School of Medicine, 3-25-8 Nishi-shimbashi, Minato-ku, Tokyo, 105-8461 Japan

**Keywords:** Unicuspid aortic valve, Infectious endocarditis, Aortic insufficiency, Nonbacterial thrombotic endocarditis

## Abstract

**Background:**

A unicuspid aortic valve is a rare congenital cardiac abnormality. Despite its uncommon finding on an initial presentation, aortic insufficiency is accompanied with unicuspid aortic valve and this might reflect the natural history of progression in the morphology of unicuspid aortic valve.

**Case presentation:**

We describe a 65-year-old Japanese man who was evaluated for endocarditis and found to have a unicuspid aortic valve concomitant with moderate aortic insufficiency, which was, owing to the lack of evidence of valve membrane destruction, independent of underlying infectious endocarditis. In addition, aortic insufficiency was progressed because of nonbacterial thrombotic endocarditis on the ventricular side, in areas of high turbulence around the heart valve.

**Conclusions:**

Our case is unusual given the unicuspid aortic valve concomitant with aortic insufficiency, which was presumably independent of underlying infectious endocarditis because of the location of the vegetation and the lack of evidence of valve destruction. Therefore, attention should be paid to a variety of complications in the setting of unicuspid aortic valve.

**Electronic supplementary material:**

The online version of this article (https://doi.org/10.1186/s13256-019-2239-9) contains supplementary material, which is available to authorized users.

## Background

A unicuspid aortic valve (UAV) is a rare congenital cardiac malformation. The occurrence of congenital UAV in adults is less well known than in children. The most common complication is aortic stenosis, which is often associated with aortic insufficiency. Cases of UAV are classified into two types: the unicommissural type and the acommissural type. Here, we report an unusual case of UAV of the unicommissural type in a man presenting with longstanding fever, eventually leading to the diagnosis of UAV concomitant with aortic insufficiency in the setting of infectious endocarditis (IE).

## Case presentation

A 65-year-old Japanese man, who had no chest pain or symptoms of syncope but was being treated for dental caries, presented with a body temperature that had increased, for reasons unknown, to 38 °C. However, because of a blood culture positive for *Streptococcus dysgalactiae*, IE was suspected. A physical examination revealed conjunctival petechiae and Janeway lesions in his bilateral upper and lower extremities. Auscultation showed a significant systolic murmur (Levine III/VI), heard best at the second right upper sternal border and radiating to his neck, but no diastolic murmur. His white blood cell count was 10,500/μL and C-reactive protein concentration was 18.1 mg/dL. Transthoracic echocardiography found an abnormal complex, which was not a tricuspid structure, on an aortic valve leaflet. The peak velocity of the left ventricular outflow tract was 4.5 m/second. Transesophageal echocardiography revealed a thick valve leaflet, a rounded vegetation with a diameter of 5 mm, a calcified surface, and border irregularity attached to the equivalent position of the aortic valve’s right coronary cusp, and mobility during diastole (Additional file 1).

**Fig. 1:**
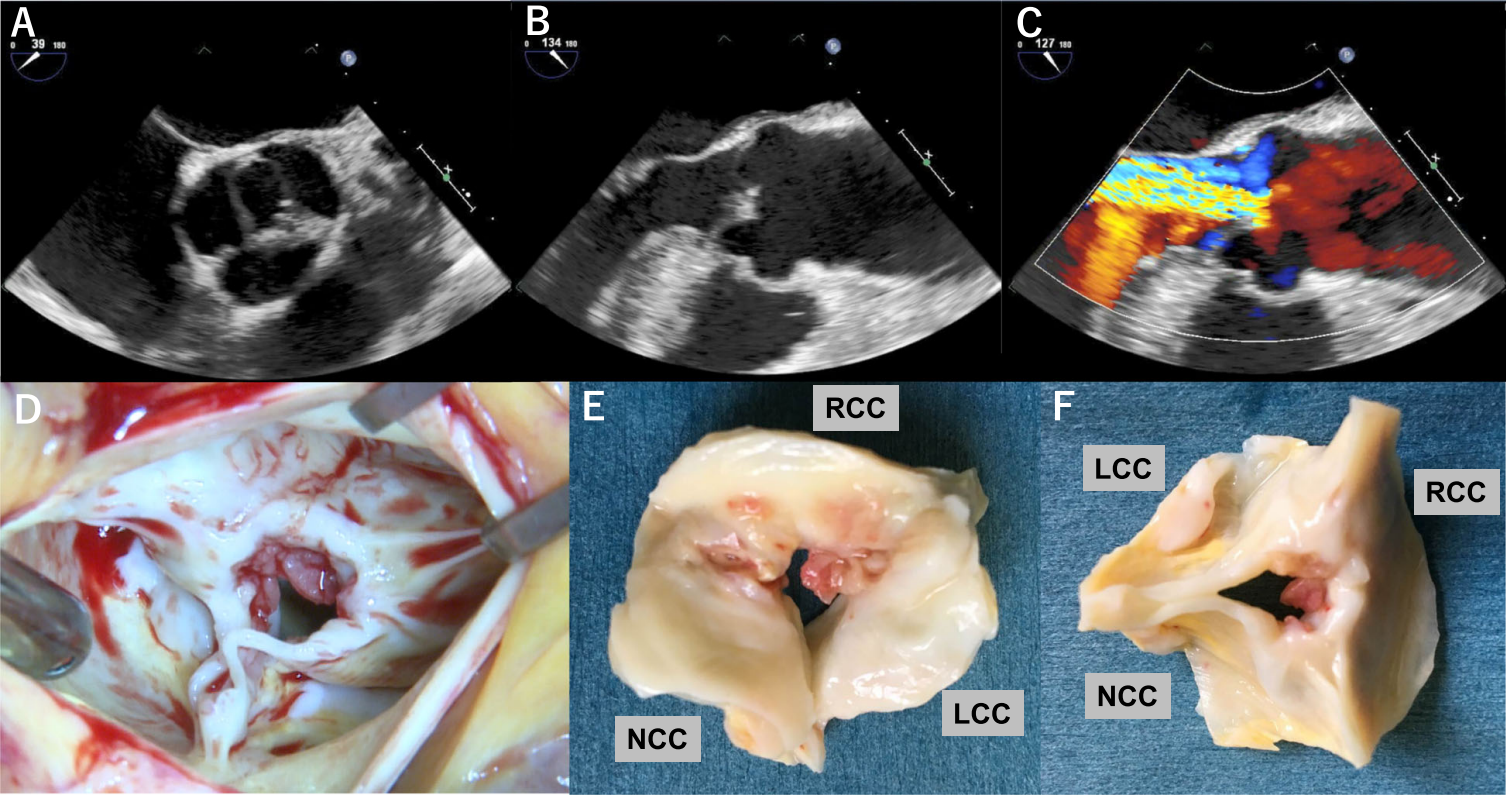
**a**–**c** Transesophageal echocardiography (**a** is midesophageal aortic valve short-axis view, **b** and **c** are midesophageal aortic valve long-axis view). Unicuspid aortic valve with a commissure at 12 o’clock and raphe at 4 o’clock and 8 o’clock (**a**). Moderate aortic regurgitant flow to posterior wall of left ventricle without any findings of valve damage, rupture of chordae tendineae and perivalvular abscess (**b**, **c**). Intraoperative finding confirmed unicuspid aortic valve with a connection of all cusps via raphe except for the commissure between left coronary cusp and noncoronary cusp, and a vegetation between left coronary cusp and right coronary cusp (**d**–**f**). *LCC* left coronary cusp, *NCC* noncoronary cusp, *RCC* right coronary cusp

On the basis of a previously reported definition [1], a UAV, with a commissure at 12 o’clock and raphae at 4 o’clock and 8 o’clock (Fig. 1a), was confirmed (Additional file 2). Moderate aortic insufficiency, with a vena contracta of 8 mm and a pressure half time of 401 milliseconds, was found with eccentric regurgitant flow to the left ventricle’s posterior wall but without valve damage, rupture of chordae tendineae, or a perivalvular abscess (Fig. 1b, c). The aortic stenosis was moderately severe with a valve area of 1.42 cm^2^. Based on the transesophageal echocardiographic findings of thickness and calcification of a valve leaflet, vegetation seems to have been formed with a chronic process, suggesting the characteristic of nonbacterial thrombotic endocarditis (NBTE). However, owing to the fever spike and positive blood culture, he was admitted to our hospital for further management of presumable IE with valvular heart disease.

Because an acute cerebral infarction was found with magnetic resonance imaging on day 1 of admission, treatment with penicillin G was started. Despite this treatment, paralysis of his right upper and lower extremities had developed owing to a newly diagnosed cerebral infarction confirmed with another magnetic resonance imaging examination on day 2. Moreover, a body temperature greater than 38 °C was found with the inflammatory markers of a white blood cell count of 13,200/uL and a C-reactive protein concentration of 10.7 mg/dL on day 3.

Because of the uncontrolled infection, the aortic valve was urgently replaced with a bioprosthetic valve on day 3. Evaluation during surgery confirmed a UAV with a connection via raphae of all cusps, except for the commissure between the left coronary cusp and the noncoronary cusp (Fig. 1d–f). With appropriate antibiotic therapy for 6 weeks after surgery, he completely recovered from IE and was discharged on day 48.

## Discussion and conclusions

A UAV is a rare congenital malformation observed with echocardiography in 0.02% of all patients [1]. In general, UAVs are classified into two types: a unicommissural type with a single commissure and an acommissural type with an opening section in the center [2]. The present case was of the unicommissural type with a commissure between the left coronary cusp and the noncoronary cusp. In addition, the aortic insufficiency occurring in this case is a common complication of UAV, with aortic stenosis being the most common complication [1]. Consistent with previously reported cases, the present case of unicommissural UAV with moderate aortic stenosis progressed slowly without symptoms, such as chest pain and syncope, before hospital admission. Furthermore, the case was diagnosed during our patient’s adulthood, which was consistent with previously reported findings. While ascending aortic dilatation, patent ductus arteriosus, and coarctation of the aorta are reported as complications of UAV because of the association of embryological abnormality, valvular heart disease is the only complication in the presented case.

Despite the little evidence that UAV and IE are associated, a study of adults with congenitally malformed aortic valves examined at autopsy found that 3 (11%) of 28 patients with UAV had the complication of IE [3]. Although the relation between UAV and IE was not examined, the study did find that 25% of patients with congenital valve malformation including UAV or a bicuspid aortic valve, had no symptoms, and that 80% of patients were men, which is consistent with the present patient being a man.

With respect to the mechanism by which IE developed in the present patient, a possibility is NBTE. Theoretically, NBTE served as a nidus for subsequent adhesion by bacteria in the blood stream and was complicated by endothelial dysfunction due to valve disease, prosthetic valve replacement, inflammatory disease, and autoimmune disease [4]. This common site implies that NBTE tends to occur in areas of high turbulence around heart valves [5]. Of interest, the vegetation within the present patient was at the ventricular side of the aortic valve and had likely been formed by NBTE and by bacteremia induced by dental caries in the setting of preexisting aortic insufficiency due to UAV. The findings of thickness and calcification of a valve leaflet were evidence of its forming as a result of a chronic process that might be compatible with NBTE. This possibility is supported by a previous report by Liu and Frishman showing that valvular vegetation, such as the present case, with no signs of infection is possibly NBTE vegetation [5]. In turn, histological findings of platelet thrombi, including fibroblastic organization, vascularization, hematoxylin bodies, and mononuclear cell infiltration, were not obtained because the explanted valve was not analyzed owing to the emergency surgery for bioprosthetic valve placement. A UAV concomitant with aortic insufficiency is reportedly caused by the valve being perforated or destroyed because of progressive IE [6].

The criteria for emergency surgery being performed for our patient were not met by the anatomical characteristics of the vegetation, such as a diameter of 5 mm. However, because infection was prolonged and followed by repetitive cerebral infarction, we decided to perform surgery. Delayed surgery may be associated with additional embolic events [7], and early surgery, within 7 days after stroke, does not significantly worsen in-hospital and 1-year mortality rates compared with delayed surgery [8]. Therefore, we believe that early surgical intervention for the present patient had the benefit of preventing newly diagnosed thromboembolic events.

This case report has the limitation of the explanted valve not being analyzed because of the emergency surgery. However, our case is unusual given the UAV concomitant with aortic insufficiency, which was presumably independent of underlying IE because of the location of the vegetation and the lack of evidence of valve destruction.

## Additional files

Additional file 1: Vegetation with a size of 5mm, calcified surface, border irregularity, and mobile during diastole. (PPTX 1230 kb)

Additional file 2: Unicuspid valve with unicommisural valve. Eccentric orifice with commissural zone at NCC-LCC and one aortic leaflet with raphe. (PPTX 1202 kb)

## Data Availability

Not applicable.

## References

[1] Novaro GM, Mishra M, Griffin BP. Incidence and echocardiographic features of congenital unicuspid aortic valve in an adult population. J Heart Valve Dis. 2003;12:674–8. 14658804

[2] Tempe DK, Garg M, Tomar AS, Dutta D, Dutta R, Singh AK. Unicuspid aortic valve: transesophageal echocardiographic features. J Cardiothorac Vasc Anesth. 2012;26:277–9. 10.1053/j.jvca.2011.01.011 21419655

[3] Roberts WC, Vowels TJ, Ko JM. Natural history of adults with congenitally malformed aortic valves (unicuspid or bicuspid). Medicine. 2012;91:287–308. 10.1097/MD.0b013e3182764b84 23117850

[4] Mann DL, Zipes DP, Libby P, Bonow RO. Braunwald’s Heart Disease: A Textbook of Cardiovascular Medicine, Single Volume. 10th ed. Philadelphia: Saunders; 2014.

[5] Liu J, Frishman WH. Nonbacterial thrombotic endocarditis: pathogenesis, diagnosis, and management. Cardiol Rev. 2016;24:244–7. 10.1097/CRD.0000000000000106 27501336

[6] Chavarria N, Kozeski G, Luo J, Lim V. Unicuspid aortic valve in a patient with *Streptococcus bovis* endocarditis. J Heart Valve Dis. 2009;18:731–2. 20099730

[7] Kang DH, Kim YJ, Kim SH, Sun BJ, Kim DH, Yun SC, *et al.* Early surgery versus conventional treatment for infective endocarditis. N Engl J Med. 2012;366:2466–73. 10.1056/NEJMoa1112843 22738096

[8] Barsic B, Dickerman S, Krajinovic V, Pappas P, Altclas J, Carosi G, *et al.* International Collaboration on Endocarditis–Prospective Cohort Study Investigators. Influence of the timing of cardiac surgery on the outcome of patients with infective endocarditis and stroke. Clin Infect Dis. 2013;56:209–17. 10.1093/cid/cis878 23074311

