# Supplementary material for: Unicuspid aortic valve concomitant with aortic insufficiency presenting with infectious endocarditis: a case report
Source: J Med Case Rep. 2019 Sep 20;13:297. doi: 10.1186/s13256-019-2239-9 (PMC6753610; doi:10.1186/s13256-019-2239-9)
Supplement: Supplementary file 1 — Vegetation with a size of 5mm, calcified surface, border irregularity, and mobile during diastole. (PPTX 1230 kb) [file 13256_2019_2239_MOESM1_ESM.pptx]

## Slide 1
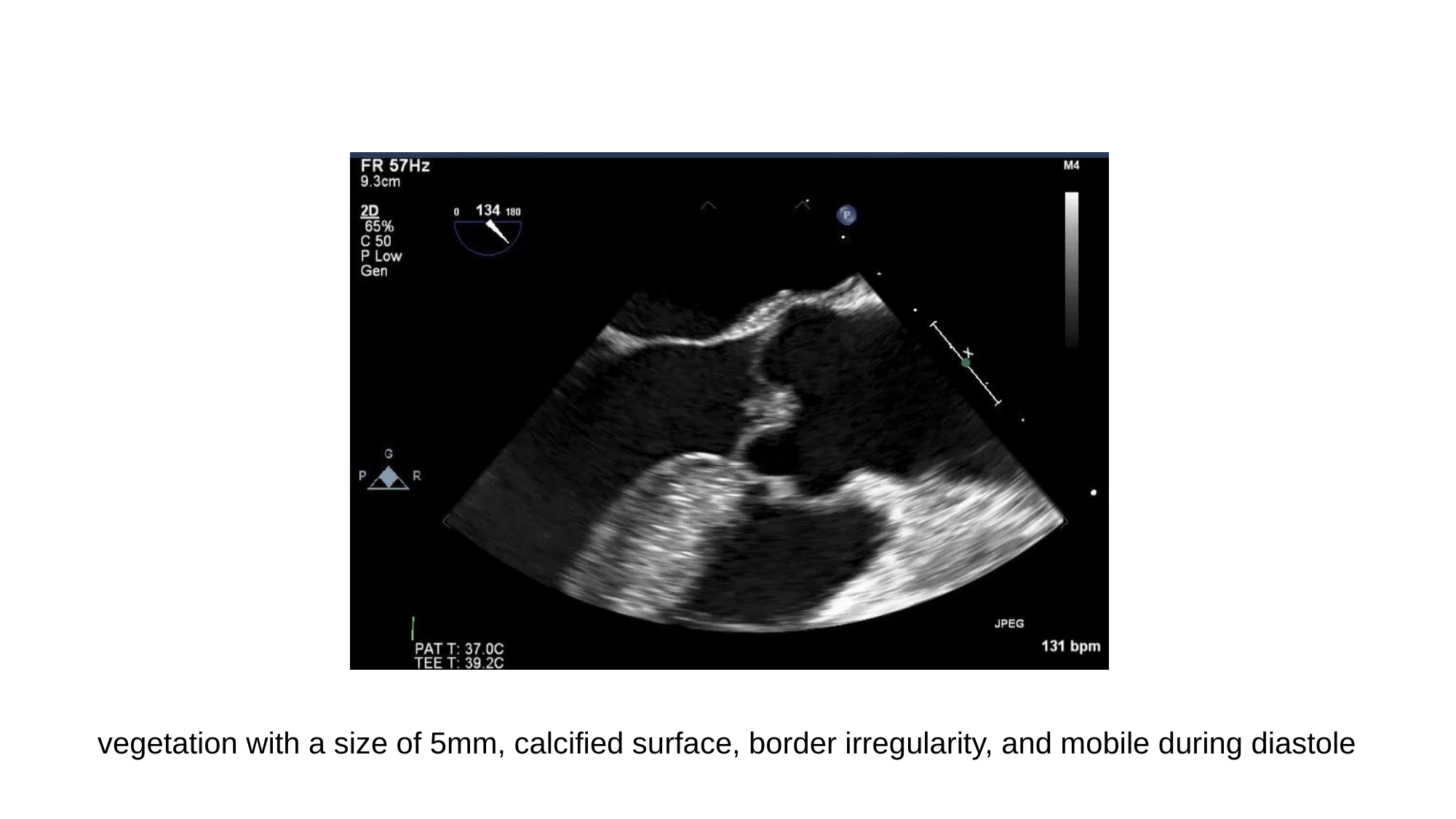

vegetation with a size of 5mm, calcified surface, border irregularity, and mobile during diastole
