# Supplementary material for: Unicuspid aortic valve concomitant with aortic insufficiency presenting with infectious endocarditis: a case report
Source: J Med Case Rep. 2019 Sep 20;13:297. doi: 10.1186/s13256-019-2239-9 (PMC6753610; doi:10.1186/s13256-019-2239-9)
Supplement: Supplementary file 2 — Unicuspid valve with unicommisural valve. Eccentric orifice with commissural zone at NCC-LCC and one aortic leaflet with raphe. (PPTX 1202 kb) [file 13256_2019_2239_MOESM2_ESM.pptx]

## Slide 1
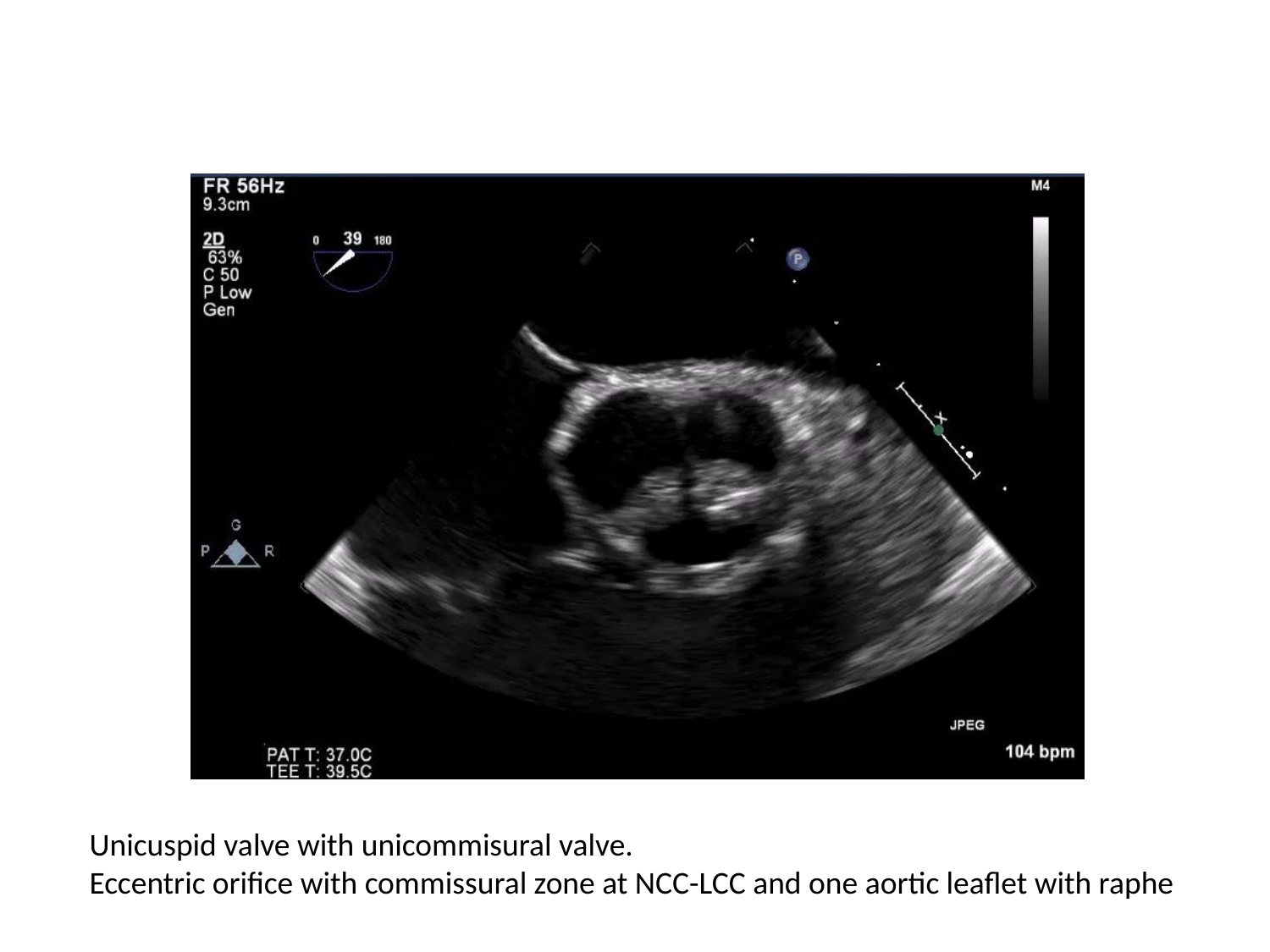

Unicuspid valve with unicommisural valve.
Eccentric orifice with commissural zone at NCC-LCC and one aortic leaflet with raphe
